# Pasture-finishing of cattle in Western U.S. rangelands improves markers of animal metabolic health and nutritional compounds in beef

**DOI:** 10.1038/s41598-024-71073-3

**Published:** 2024-08-30

**Authors:** Nikia Evans, Jennifer Cloward, Robert E. Ward, Herman A. van Wietmarschen, Nick van Eekeren, Scott L. Kronberg, Frederick D. Provenza, Stephan van Vliet

**Affiliations:** 1 School of Medicine, Oregon Health & Science University, Portland, OR 97239 USA; 2 Department of Nutrition, Dietetics, and Food Sciences, Center for Human Nutrition Studies, Utah State University, Logan, UT 84322 USA; 3 Duke Molecular Physiology Institute, Duke University Medical Center, Durham, NC 27701 USA; 4 Louis Bolk Institute, Bunnik, 3981 AJ The Netherlands; 5 Northern Great Plains Research Laboratory, USDA-Agricultural Research Service, Mandan, ND 58554 USA; 6 Department of Wildland Resources, Utah State University, Logan, UT 84322 USA

**Keywords:** Pasture-finished, Grass-fed, Beef, Meat, Omega-3 fats, Phytochemical, Nutrition, Metabolic health, Metabolomics, Physiology, Agroecology, Metabolomics

## Abstract

As environmental and health concerns of beef production and consumption mount, there is growing interest in agroecological production methods, including finishing beef cattle on pastures with phytochemically diverse grasses, forbs, and/or shrubs. The goal of this metabolomics, lipidomics, and fatty acid methyl ester profiling study was to compare meat (*pectoralis profundus*) of Black Angus cattle from two commercial US beef finishing systems (pasture-finished on Western U.S. rangeland; *n* = 18 and grain-finished in a Midwest U.S. feedlot; *n* = 18). A total of 907 out of 1575 compounds differed in abundance between pasture-finished and grain-finished beef samples (all, false discovery rate adjusted *P* < 0.05). Pasture-finished beef contained higher levels of phenolic antioxidants (2.6-fold), alpha-tocopherol (3.1-fold), nicotinate/vitamin B_3_ (9.4-fold), choline (1.2-fold), myo-inositol (1.8-fold), and omega-3 fatty acids (4.1-fold). Grain-finished beef contained higher levels of gamma-tocopherol (14.6-fold), nicotinamide/vitamin B_3_ (1.5-fold), pantothenate/vitamin B_5_ (1.3-fold), and pyridoxine/vitamin B_6_ (1.3-fold); indicating that feeding some grain (by-products) could be beneficial to increase levels of certain B-vitamins. Pasture-finished beef samples also displayed lower levels of oxidative stress (homocysteine, 0.6-fold; and 4-hydroxy-nonenal-glutathione, 0.4-fold) and improved mitochondrial function (1.3-fold) compared to grain-finished animals. Two potential metabolites of fluoroquinolone antibiotics, 2,8-quinolinediol and 2,8-quinolinediol sulfate, were only observed in grain-finished beef, though the source remains unknown. While pasture-finished cattle displayed improved markers of metabolic health and concentrated additional, potentially health-promoting compounds in their meat, our findings should not be interpreted as that grain-finished beef is unhealthy to consume. Randomized controlled trials in humans are required to further assess whether observed differences between pasture-finished and feedlot-finished beef have an appreciable effect on human health.

## Introduction

Beef, irrespective of finishing practice, contributes several essential nutrients to the US diet, including protein, zinc, iron, and various B-vitamins^1^. As consumers have become increasingly conscious of potential animal welfare issues, environmental impacts, and health concerns surrounding the consumption of red meat^2^, there has been a growing interest in pasture-finished (grass-finished) beef. The U.S. pasture-finished beef market experienced rapid growth over the last decade^3^, and is expected to continue to grow during the next decade^4^. Pasture-finishing of livestock is often perceived as more ethical, healthy, and environmentally sustainable, although variations and trade-offs exist amongst both feedlot (grain-finished) and pastured (grass-finished) systems^5,6^. Nonetheless, growing consumer and producer interest in pasture-finished beef products has increasingly led to questions about potential nutritional differences between pasture-finished and feedlot-finished beef.

Previous studies have found that pasture-finished beef has a higher omega-3 fatty acid and total antioxidant/phenolic content when compared to grain-finished beef^5,7, 8^. Novel evidence indicates that pasture-finishing of ruminants results in greater accumulation of phytochemicals—phenolics, terpenes, carotenoids and other antioxidant compounds—in meat, which appears directly related to the phytochemical richness of the forage/feed consumed by ruminants^9,10^. Further, studies of cattle, goats, and sheep on pasture generally report improved markers of animal welfare, especially when ruminants have the ability to select from a diverse mixture of grasses, forbs, and shrubs, and are able to engage in innate behavior^11–14^. Despite differences between feedlot- and pasture-finished beef regarding total fatty acids, total phenolics/antioxidants, and animal welfare markers, few studies have constructed a comprehensive metabolic profile of beef from these two systems.

Advances in omics-based technologies have allowed for a broader and deeper understanding of the nutritional and metabolic components in food (often referred to as the “dark matter of nutrition”)^15,16^. By integrating multiple omics-based approaches with fatty acid methyl ester analysis, the goal of this work was to profile over 1500 compounds, across multiple metabolic classes, to provide comprehensive insight into how two commercial U.S. beef finishing modes (pasture-finished on Western U.S. rangeland and grain-finished in a Midwest U.S. feedlot system) impacted metabolites related to animal metabolic health and meat nutritional composition.

## Results

### Statistical summary and significantly altered biochemicals

Untargeted metabolomics analysis of the beef samples identified 536 known compounds (named biochemicals) and 41 unknown compounds (unnamed biochemicals); for a total of 577 identified compounds (Supplementary Table 1). Of the 577 total compounds identified, 377 (65%) differed in abundance (*P* < 0.05) between pasture (bf_pasture)- and grain-finished (bf_grain) beef samples (Table 1). Lipidomics profiling found that 530 (53%) out of 998 identified compounds differed in abundance between pasture- and grain-finished beef samples (*P* < 0.05) (Supplementary Table 2).

**Table 1:** Untargeted metabolomics and lipidomics profiling comparisons. Bold values indicate higher in pasture-finished, italic values indicate higher in grain-finished.

	Metabolomics	Lipidomics
Welch’s two-sample t-test	Bf_Pasture Bf_Grain	Bf_Pasture Bf_Grain
Total biochemicals identified	578	998
Total metabolites, *p* ≤ 0.05	377 (65%)	530 (53%)
Biochemicals (**↑│↑**)	**286**│*91*	**307**│*223*
Total metabolites, 0.05 > P ≤ 0.10	26 (4%)	86 (9%)
Biochemicals (**↑│↑**)	**18**│*8*	**47**│*36*

**Fig. 1:**
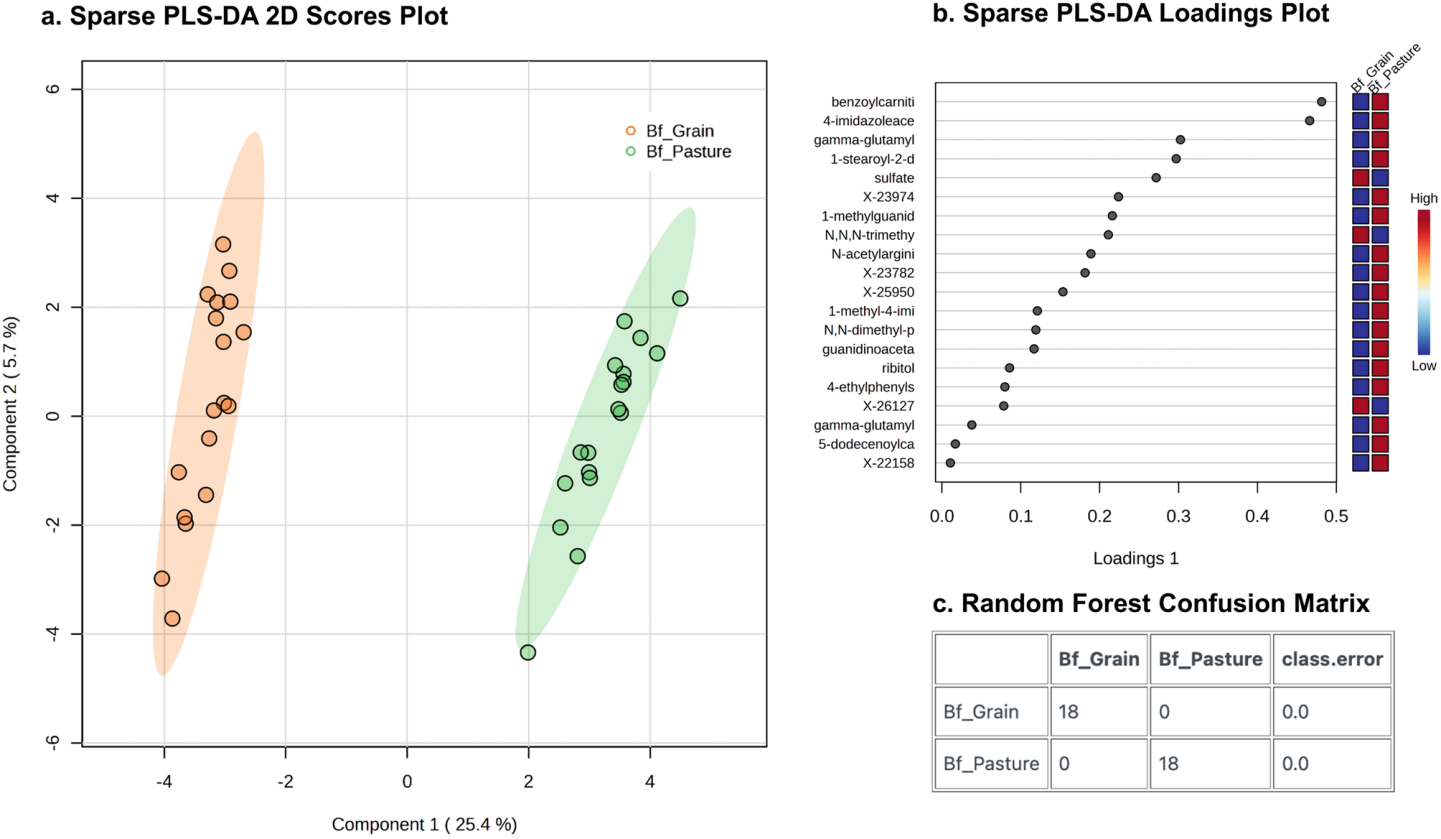
(**a**) Sparse partial least-squares discriminant analysis (sPLS-DA) of the untargeted metabolite data for the beef samples found strong separation between pasture- and grain-finished samples along Component 1 (explaining 25.4% of the variation) and Component 2 (explaining 5.7% of the variation). (**b**) sPLS-DA Loadings Plot of Component 1, which highlights the top 20 metabolites important for the separation between pasture- and grain-finished beef, ranked in order of increasing importance from bottom to top on the y-axis. (**c**) Random forest confusion matrix showed a predictive value of 100% (0.0 class error).

Sparse Partial Least-Squares Discriminant Analysis (sPLS-DA) of metabolites found strong separation between pasture- and grain-finished samples along Component 1 (explaining 25.4% of the variation; Fig. 1A), with the top 20 components from Loading Plot 1 highlighted (Fig. 1B). The random forest confusion matrix had a predictive value of 100% (0.0 class error), which indicates that the differences between the pasture-finished and grain-finished samples are not due to random chance (Fig. 1C).

**Fig. 2:**
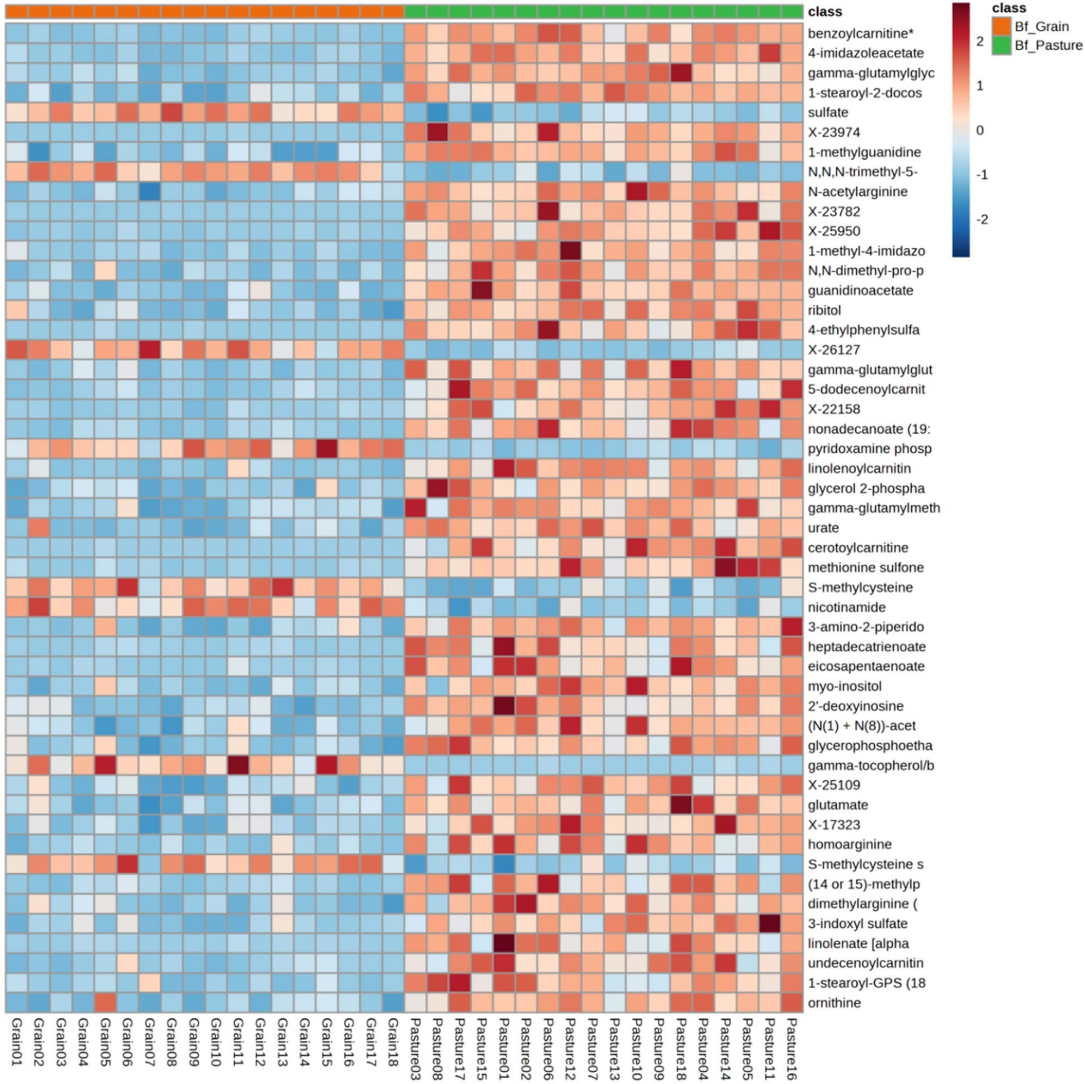
Heatmap of top 50 metabolites ranked according to the P-values (lowest to highest) obtained from the Welch’s two-sample* t*-test (all compounds have a value of *P* < 0.05). Red (intensity ranges from 0 to 1.5) means higher abundance of the corresponding metabolite, whereas blue means lower abundance (intensity ranges from − 2 to 2). The numbers below the heatmap represent individual samples (Grain01 to 18 and Pasture01 to 18 respectively; n = 18 for each group). Metabolites in pasture-finished and grain-finished diets were compared by the Wilcoxon rank sum test with Benjamini–Hochberg adjusted p values at 5% (*P* < 0.05).

**Fig. 3:**
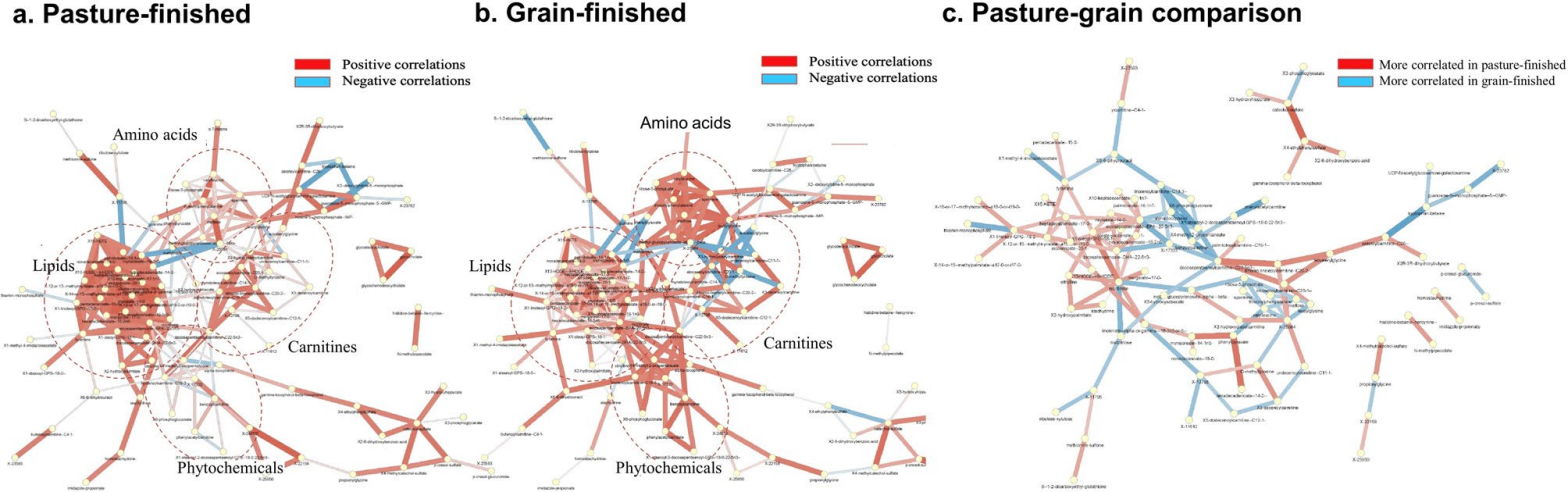
Network visualization of Spearman rank correlations from log transformed data with values > 0.70 and <  − 0.70 calculated of the variables with PCA loadings > 0.60 and <  − 0.60 for the pasture-finished (**a**) and grain-finished (**b**) groups, and as a comparison (**c**) with pasture-finished data subtracted from the grain-finished data. Red connecting lines represent positive correlations with darker red representing higher correlations. Blue connecting lines represent negative correlations, with darker blue representing higher correlations. The thickness of the connecting lines represents strength of the correlation. Groups of related metabolites are marked by dashed circles.

The following super pathways and their respective metabolites were found to contribute to the differences between pasture-finished and grain-finished samples; *lipid metabolites* such as alpha-linolenic acid (ALA) and eicosapentaenoic acid (EPA), *phytochemicals/xenobiotics* such as benzoylcarnitine, 4-ethylphenylsulfate, and phenol sulfate, *vitamins* including gamma/beta tocopherol (a precursor of Vitamin E) and niacin (Vitamin B_3_), and various *amino acid metabolites* including *S*-methylmethionine and homocysteine, which are involved in the *S*-adenosylmethione (SAM) cycle (Fig. 2; Supplementary Table 1). ChemRICH pathway analysis confirmed these findings and identified that 50 out of 57 metabolic pathways differed between pasture-finished and grain-finished samples, with (i) *phenolic metabolism*, (ii) *leucine, isoleucine and valine metabolism*, and (iii) *urea cycle; arginine and proline metabolism* emerging as the dominant pathways altered in response to finishing mode (Table 2). Network analysis of spearman rank correlations (Fig. 3) indicated differences in connectivity between omega-3 fatty acids, phytochemicals, carnitines, and tocopherols between pasture-finished and grain-finished samples.

**Table 2:** Differences in metabolite sub pathways with from beef finished on pasture or on grain. Analyzed using chemical similarity enrichment analysis software (ChemRICH; https://chemrich.idsl.me/home). Pathways with > 5 compounds are displayed.

Sub pathway	Cluster size	*P*-value	FDR	Key compound	Altered compounds	↑Pasture	↑Grain
Phenolic metabolism	32	< 0.001	< 0.001	4-Ethyl phenyl sulfate	25	21	4
Leucine, isoleucine and valine metabolism	25	< 0.001	< 0.001	2-Methyl butyryl carnitine (C5)	12	7	5
Urea cycle; arginine and proline metabolism	20	< 0.001	< 0.001	*N*-Acetyl arginine	14	14	0
Methionine, cysteine, SAM and taurine metabolism	19	< 0.001	< 0.001	Methionine sulfone	10	6	4
Lysine metabolism	17	< 0.001	< 0.001	*N*,*N*,*N*-trimethyl-5-aminovalerate	12	10	2
Histidine metabolism	16	< 0.001	< 0.001	1-Methyl-4-imidazole acetate	9	5	4
Dipeptide	16	< 0.001	< 0.001	Prolylglycine	8	2	6
Long chain polyunsaturated fatty acid (n3 and n6)	12	< 0.001	< 0.001	Heptadecatrienoate (17:3)	10	9	1
Purine metabolism, hypoxanthine	12	< 0.001	< 0.001	2′-Deoxy inosine	7	6	1
Gamma-glutamyl amino acid	11	< 0.001	< 0.001	Gamma-glutamyl glutamate	11	11	0
Glutamate metabolism	11	< 0.001	< 0.001	Glutamate	9	9	0
Fatty acid, dicarboxylate	11	0.001	0.002	Maleate	6	4	2
Glutathione metabolism	10	0.000	0.000	Ophthalmate	10	7	3
Fatty acid metabolism (acyl carnitine, monounsaturated)	10	0.000	0.000	5-Dodecenoyl carnitine (C12:1)	8	4	4
Pyrimidine metabolism, uracil containing	10	0.000	0.000	5-Methyluridine (ribothymidine)	7	6	1
Fatty acid metabolism (acyl carnitine, polyunsaturated)	10	0.000	0.000	Linolenoylcarnitine (C18:3)	7	5	2
Phospholipid metabolism	10	0.000	0.000	Glycerophosphoethanolamine	7	5	2
TCA cycle	10	0.000	0.000	Aconitate [cis or trans]	6	4	2
Tyrosine metabolism	10	0.001	0.001	Tyramine	6	3	3
Nicotinate and nicotinamide metabolism	10	0.006	0.007	Nicotinate	5	2	3
Tryptophan metabolism	9	0.000	0.000	3-Indoxyl sulfate	7	6	1
Fatty acid, monohydroxy	9	0.000	0.000	13-HODE + 9-HODE	6	4	2
Glycolysis, gluconeogenesis, and pyruvate metabolism	9	0.000	0.000	3-Phospho glycerate	5	3	2
Alanine and aspartate metabolism	9	0.000	0.000	Aspartate	6	5	1
Pentose metabolism	9	0.002	0.003	Ribitol	5	4	1
Glycine, serine and threonine metabolism	9	0.005	0.007	Glycine	5	5	0
Long chain saturated fatty acid	8	0.000	0.000	Nonadecanoate (19:0)	8	8	0
Aminosugar metabolism	8	0.000	0.000	Glucuronate	6	4	2
Purine metabolism, adenine containing	8	0.000	0.001	N6-succinyl adenosine	5	5	0
Fatty acid metabolism (acyl carnitine, long chain saturated)	8	0.061	0.069	Cerotoyl carnitine (C26)	3	3	0
Long chain monounsaturated fatty acid	7	0.000	0.000	10-Nonadecenoate (19:1n9)	7	7	0
Polyamine metabolism	7	0.000	0.000	(N(1) + N(8))-Acetyl spermidine	5	4	1
Fatty acid metabolism (acyl carnitine, hydroxy)	7	0.017	0.022	3-Hydroxy oleoylcarnitine	3	1	2
Fatty acid metabolism (acyl carnitine, medium chain)	6	0.000	0.000	cis-3,4-Methyleneheptanoylcarnitine	6	4	2
Lysophospholipid	6	0.000	0.000	1-Linolenoyl-GPG (18:3)	5	5	0
Endocannabinoid	6	0.000	0.000	Oleoyl ethanolamide	4	1	3
Phenylalanine metabolism	6	0.001	0.001	Phenylpyruvate	3	1	2
Purine metabolism, guanine containing	6	0.002	0.002	Guanosine 5′-monophosphate (5′-GMP)	4	2	2
Fatty acid metabolism (also BCAA metabolism)	6	0.006	0.008	Propionylcarnitine (C3)	3	2	1
Ascorbate and aldarate metabolism	5	0.000	0.001	Gulonate*	4	4	0
Pyrimidine metabolism, cytidine containing	5	0.012	0.016	2′-Deoxycytidine 5′-monophosphate	2	2	0
medium chain fatty acid	5	0.020	0.024	Laurate (12:0)	2	2	0
vitamin B6 metabolism	5	0.039	0.044	Pyridoxamine phosphate	2	0	2
Fructose, mannose and galactose metabolism	4	0.004	0.006	Mannitol/sorbitol	3	3	0

#### Lipid and fatty acid metabolites

The fatty acid content of pasture- and grain-finished beef samples (Table 3) is presented as % of total fatty acids. Twelve of twenty measured individual fatty acids were different between pasture-finished and grain-finished beef (all, *P* < 0.05). Among the polyunsaturated fatty acids (PUFAs), pasture-finished beef contained 4.1-fold higher amounts of total omega-3 fatty acids compared to grain-finished beef. More specifically, pasture-finished beef contained 3.5-fold and 2.6-fold higher amounts of eicosapentaenoic (EPA) and docosapentaenoic acid (DPA), respectively, compared to grain-finished beef. The omega-6 fatty acid linoleic acid was 1.6-fold higher in grain-finished beef compared to pasture-finished beef. As a result, the ratio of omega-6 to omega-3 fatty acids was significantly higher in grain-finished (10.85 ± 7.0) compared to pasture-finished samples (1.58 ± 0.25) (*P* < 0.05). Similarly, long-chain monounsaturated fatty acids, such as oleate and palmitoleate, and long-chain saturated fatty acids, such as arachidic acid, were 1.7- to 4.1-fold higher in pasture-finished beef. Finally, total triacylglycerols (TAGs) were 0.5-fold lower in pasture-finished samples, while individual long-chain acyl carnitines, which transport PUFAs to the mitochondria for β-oxidation, were 1.5- to 9-fold higher in pasture-finished samples (all, *P* < 0.05; Supplemental Table 2).

**Table 3:** Fatty acid content of pasture- and grain-finished beef (% fatty acids).

Carbon #	Fatty acid	Pasture-finished Mean ± SD	Grain-finished Mean ± SD
C14:0	Myristic acid	2.64 ± 0.32	2.45 ± 0.25
C15:0*	Pentadecanoic acid	0.70 ± 0.08	0.38 ± 0.05
C16:0	Palmitic acid	23.77 ± 1.22	2.396 ± 2.02
C16:1n7t*	Palmitoleic acid (trans)	0.74 ± 0.13	0.39 ± 0.22
C16:1n7c	Palmitoleic acid (cis)	4.28 ± 0.72	4.38 ± 1.36
C17:0	Heptadecanoic acid	1.37 ± 0.12	1.01 ± 0.18
C18:0*	Stearic acid	16.47 ± 2.20	10.47 ± 2.20
C18:1t9	Elaidic acid	0.28 ± 0.13	0.64 ± 0.93
C18:1n9*	Oleic acid	34.98 ± 2.68	39.07 ± 2.09
C18:1n7*	Vaccenic acid	1.43 ± 0.16	2.15 ± 0.38
C18:1n-7t*	Vaccenic acid (trans)	2.62 ± 0.66	0.89 ± 1.08
CLA, cis-9, trans-11*	Rumenic acid	0.61 ± 0.21	0.36 ± 0.17
C18:2n6*	Linoleic acid	1.99 ± 0.38	3.11 ± 1.37
C18:3n3*	Alpha linolenic acid (ALA)	0.96 ± 0.06	0.17 ± 0.01
C20:0*	Arachidic acid	0.22 ± 0.03	0.06 ± 0.02
C20:1n9*	Eicosenoic acid	0.18 ± 0.02	0.28 ± 0.07
C20:3n6	Dihomo-gamma-linolenic acid	0.18 ± 0.03	0.18 ± 0.07
C20:4n6	Arachidonic acid	0.59 ± 0.15	0.57 ± 0.22
C20:5n3*	Eicosapentaenoic acid (EPA)	0.31 ± 0.11	0.09 ± 0.05
C22:5n3*	Docosapentaenoic acid (DPA)	0.42 ± 0.10	0.16 ± 0.10
C24:1n9*	Nervonic acid	0.07 ± 0.05	0.15 ± 0.07
n6-n3 ratio*	Omega-6: Omega-3	1.58 ± 0.25	10.85 ± 7.00

*Indicates a significant difference (*P* < 0.05) between fatty acid concentrations in pasture-and grain-finished beef. *SD* standard deviation.

#### Carbohydrate and energy metabolites

**Fig. 4:**
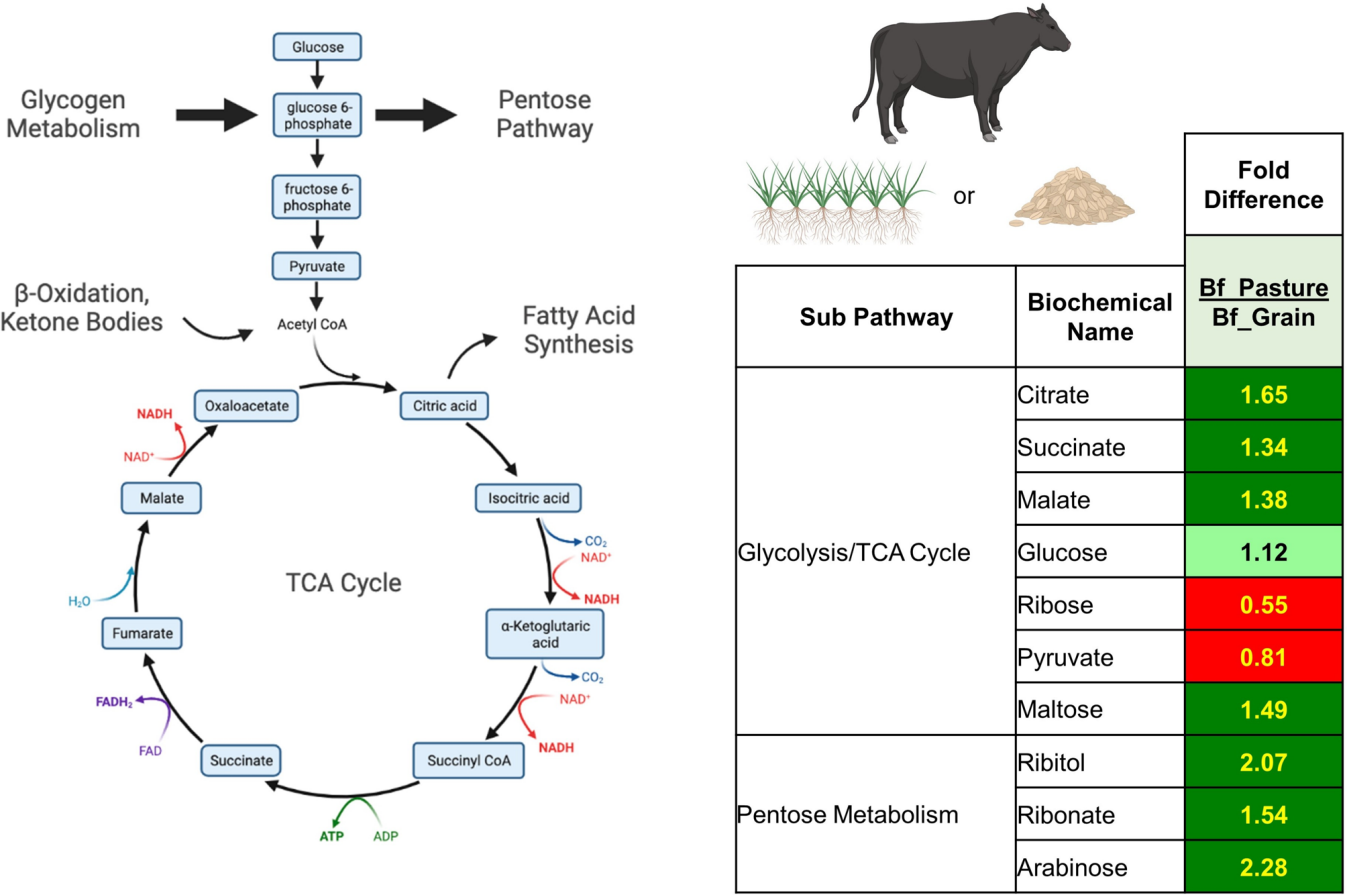
TCA Cycle Intermediates including citrate, succinate, and malate were elevated by 1.3- to 1.6-fold in pasture-finished beef samples (all, *P* < 0.05). Although no differences were found for glucose (*P* > 0.05), the levels of ribose and pyruvate were increased in grain-finished beef samples (*P* < 0.05). Maltose, an intermediate of glycolytic metabolism, along with ribitol, ribonate, and arabinose, intermediates of pentose metabolites, were 1.5–2.3 fold elevated in pasture-finished beef samples (all, *P* < 0.05). Values on figures are expressed as arbitrary units (AU).

Tricarboxylic acid (TCA) cycle intermediates including citrate, succinate, and malate were, on average, 1.3- to 1.6-fold higher in pasture-finished beef samples (all,* P* < 0.05) (Fig. 4; Supplementary Table 1), indicating higher mitochondrial muscle fatty acid oxidation, and therefore, a more oxidative phenotype in pasture-finished animals. No differences were found for glucose (*P* > 0.05), while pyruvate, a major intermediate in glycolytic metabolism was 1.2-fold higher in grain-finished beef samples (*P* < 0.05). Ribitol, ribonate, and arabinose, which are pentose metabolites, were 1.5- to 2.3-fold higher in pasture-finished beef samples (all, P < 0.05; Supplementary Table 1), indicating greater metabolic activity of the pentose phosphate pathway in pasture-finished animals.

#### Amino acid/nucleotide metabolites

**Fig. 5:**
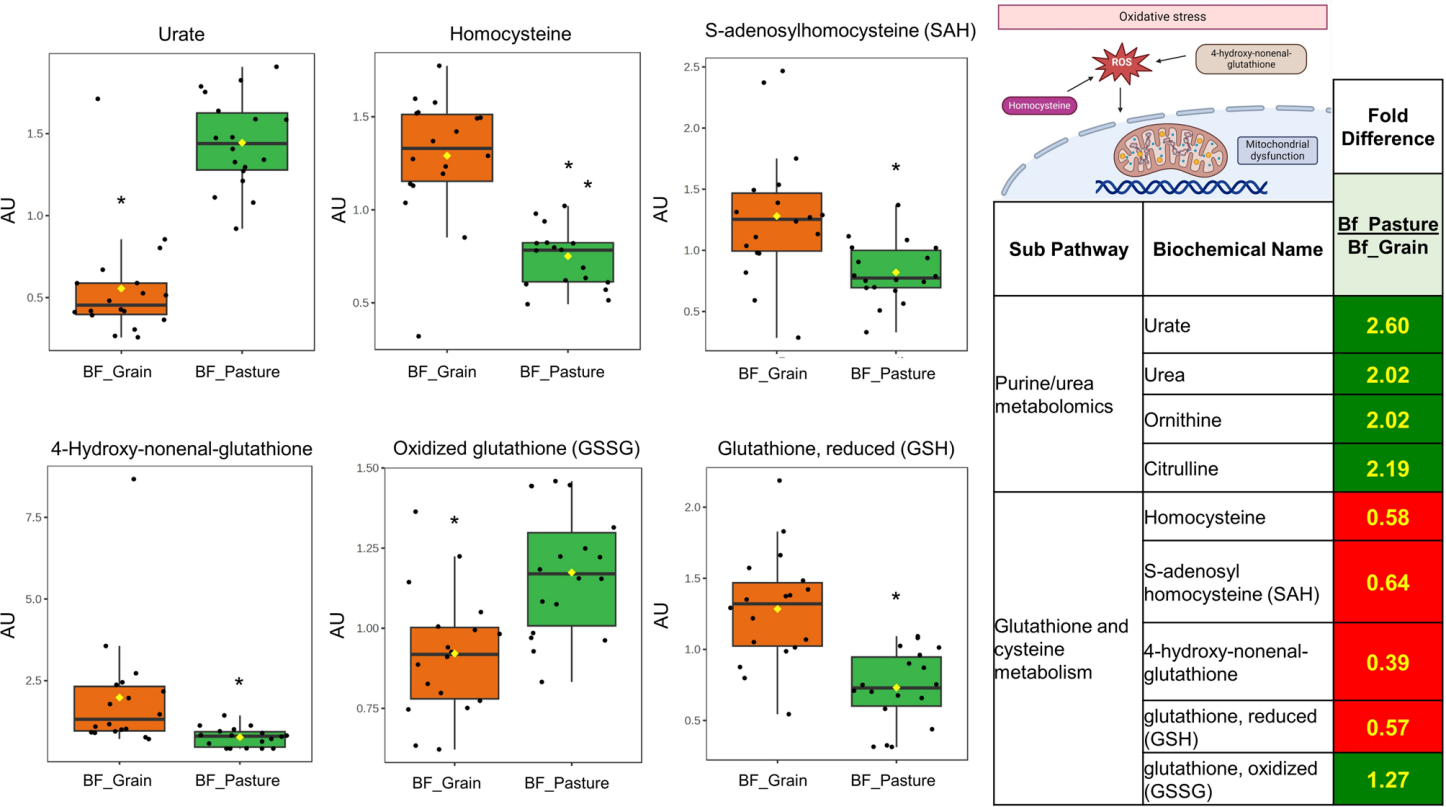
Urate, a major intracellular antioxidant, was elevated by 2.6-fold in pasture-finished samples while 4-hydroxynonenal glutathione, an advanced lipoxidation end product, was elevated by 2.6-fold in grain-finished samples. Homocysteine was increased 1.7-fold and S-adenosylhomocysteine was increased by 1.5-fold in grain-finished beef (all, *P* < 0.05). Higher levels of oxidized glutathione and lower levels of reduced glutathione were observed in pasture-finished beef samples compared to grain-finished beef samples. Values on figures are expressed as arbitrary units (AU).

Urea cycle metabolites were, on average, 2- to 2.9-fold higher in pasture-finished beef samples (all, *P* < 0.05) (Supplementary Table 1). Carnitine and carnitine metabolites were 1.2- to 1.5-fold higher in pasture-finished (all, *P* < 0.05). Urate (an intracellular antioxidant) was elevated 2.6-fold in pasture-finished samples while 4-hydroxynonenal glutathione (a lipid peroxidation product related with various pathologies and disease states), was elevated 2.6-fold in grain-finished samples (Fig. 5) (all, *P* < 0.05). Homocysteine and *S*-adenosylhomocysteine (non-proteinogenic α-amino acid metabolites potentially indicative of oxidative stress) were increased 1.5 and 1.7-fold in grain-finished beef, respectively (all, *P* < 0.05). Finally, we observed 1.27-fold higher levels of oxidized glutathione and 1.75-fold lower levels of reduced glutathione in pasture-finished beef (all, *P* < 0.05) (Fig. 4) (Supplementary Table 1).

#### Vitamins and cofactors

Several vitamin metabolites differed between pasture-finished and grain-finished beef (Supplementary Table 1). Among these, alpha-tocopherol (a Vitamin E precursor), niacin (a form of Vitamin B_3_), choline, and dehydroascorbate (a product of Vitamin C metabolism) were 3-, 9.4-, 1.2-, and 1.6-fold higher in pasture-finished beef, respectively (all, *P* < 0.05). Alternately, pantothenate (Vitamin B5), gamma/beta tocopherol (a Vitamin E precursor), and nicotinamide (a form of Vitamin B_3_) were 1.3-, 14.6-, and 1.6-fold higher in grain-finished beef, respectively (all, *P* < 0.05).

#### Phytochemicals/xenobiotics

Total phytochemicals were 2.6-fold higher in pasture-finished beef (*P* < 0.05) (Supplementary Table 1). Specifically, hippurate and its downstream metabolites catechol sulfate and 4-ethylphenyl sulfate were 1.9- to 7.1-fold higher in pasture-finished beef (all, *P* < 0.05). Cinnamoylglycine, a glycine conjugate of cinnamic acid, was 1.4-fold higher, while *n*-methylpipecolate, a metabolite of coumaric acid, was 4.9-fold higher in pasture-finished beef (all, *P* < 0.05). Other phenolic metabolites elevated in pasture-finished beef included p-cresol sulfate, as well as stachydrine and homostachydrine (two major compounds derived from plants in the *Stachys genus*). Furthermore, 2,8-quinolinediol and 2,8-quinolinediol sulfate, potential metabolites of antibiotic fluoroquinolones, were detected in 97% of the grain-finished beef samples, but not pasture-finished beef samples. We also found piperidine, an alkaloid commonly found in hay, to be 2.6-fold higher in grain-finished beef samples (all, *P* < 0.05).

## Discussion

By integrating multiple omics-based approaches, we found substantial differences in compounds related to animal metabolic health and meat nutritional composition in beef samples from two US finishing modes (pasture-finished in Western U.S. rangelands and grain-finished in a Midwest U.S. feedlot system). A total of 907 out of 1575 profiled compounds (all, *P* < 0.05) differed between pasture-finished and grain-finished beef samples, with key involvement of metabolic classes such as lipid metabolites, carbohydrate metabolites, amino acid/nucleotide metabolites, vitamins and cofactors, and phytochemicals. Overall, pasture-finishing of cattle in Western U.S. rangelands concentrated more compounds considered to be health-promoting, including very long-chain fatty acids, phytochemical antioxidants, niacin, and long-chain acyl carnitines, while benefitting animal metabolic health as observed by lower levels of oxidative stress, improved mitochondrial health, and lower levels of advanced glycation end-products.

### Lipid metabolites

We examined a variety of lipid metabolites relevant to human health including very long-chain polyunsaturated fatty acids (PUFAs) and saturated fatty acids (SFAs), and triacylglycerols and long-chain acyl carnitines. Regarding PUFAs, we observed that pasture-finished beef contained 4.1-fold higher amounts of omega-3 fatty acids, including alpha-linolenic acid (C18:3*n*3; ALA), eicosapentaenoic (C20:5*n*3; EPA), and docosapentaenoic acid (C22:5*n*3; DPA). In contrast, linoleic acid, a common omega-6 fatty acid found in grain, was 1.6-fold higher in grain- than pasture-finished beef samples. As a result, the average ratio of omega-6 to omega-3 fatty acids was significantly lower in pasture-finished (*n*-6:*n*-3 ratio; 1.58) compared to grain-finished samples (*n*-6:*n*-3 ratio; 10.85). While ruminant meat is sometimes considered a negligible source of omega-3 fatty acids, randomized controlled trials have found that consuming pasture-finished beef or lamb in the habitual diet of healthy individuals can improve blood omega-3 fatty acids (specifically, EPA and DPA) after several weeks of regular consumption of amounts ranging from 69 to 500 g/day^17,18^. Population-based studies in Ireland, the United Kingdom and Australia, where pasture-finished meat consumption is more common, further indicate potential meaningful contributions of pasture-finished meat to overall dietary long-chain omega-3 fatty acid intake^19–21^. For example, Howe et al., 2006 modeled that beef and lamb contributed 68 mg of combined EPA, DPA, and DHA (28% of total long-chain omega 3 intake) to the diet of Australian adults^20^. Modeling scenarios in UK adults indicate that 16.5–66.4% of recommended DHA + EPA intakes can be met by consuming between 14 to 70 g of pasture-finished beef per day^19^. This information is noteworthy, as higher dietary intakes of omega-3 fatty acids are associated with improved cardiometabolic health^22–24^, and part of it can potentially be met by consuming pasture-finished red meat.

Besides the effects of pasture-finishing on enriching long-chain PUFAs in meat, long-chain saturated fatty acids—nonadecanoate (C19:0), arachidate (C20:0), and behenate (C22:0)—were also enriched in pasture-finished beef (Table 3). Higher circulating levels of very long-chain SFAs (C19:0 and longer) are associated with decreased risk of diabetes and cardiovascular disease in population-based studies^19,20, 25^. Also noteworthy were the 1.8-fold higher levels of pentadecanoic acid (C15:0) in pasture-finished meat. Higher dietary intakes and circulating levels of pentadecanoic acid have been associated with improved human cardiovascular health^24^. While studies have found that regular intake of pasture-finished beef can increase blood omega-3 levels^17,18^, it remains to be determined if pasture-finished beef can also increase circulating levels of very long-chain SFAs in the blood of consumers. We also found that stearic acid (C18:0) was 1.6-fold higher in the pasture-finished meat, while no differences were found in palmitic (C16:0) and myristic acid (C14:0) content between the pasture-finished and grain-finished sample. Stearic acid, relative to other saturated fatty acids such as palmitic and myristic acid, is considered less atherogenic^26,27^.

Finally, we found that long-chain acyl carnitines (C18–C26) were 1.8-fold higher in pasture-finished samples. Long-chain acyl carnitines transport long-chain fatty acids to the mitochondria for β-oxidation^28^. Together with the finding that various tricarboxylic acid (TCA) cycle metabolites were elevated in the pasture-finished beef (described below), we consider this indicative of increased oxidative metabolism in pasture-finished animals.

### Carbohydrate and energy metabolites

The profile of energy metabolites detected in beef samples serves to characterize capacity for oxidative and glycolytic metabolism in cattle. We observed that TCA cycle intermediates including citrate, succinate, and malate were 1.3- to 1.6-fold higher in pasture-finished beef samples. While no differences were found for glucose, the levels of ribose and pyruvate were increased 1.8-fold and 1.2-fold in grain-finished beef samples indicating a greater reliance on glycolytic metabolism^29^. Ribitol, ribonate, and arabinose, intermediates of pentose metabolites, were elevated 1.5- to 2.3-fold in pasture-finished beef samples. The pentose phosphate pathway, parallel to glycogen metabolism, is essential for red blood cells to ameliorate oxidative damage by the regeneration of glutathione and provides the main source of energy for red blood cells^30^. Overall, our findings indicate increased oxidative metabolism in the pasture-finished animals, which is in line with previous muscle transcriptomic/metabolic profiling work on pasture-finished cattle^10^. We consider factors including higher levels of physical activity in the pasture-finished cattle, and intakes of forages rich in long-chain fatty acids such as ALA as a likely explanation for those observations^31,32^. Furthermore, higher intakes of grain in the feedlot-finished animals and less movement likely explains their higher reliance on glycolytic metabolism^14,33^. A limitation of this study is that physical activity was not measured and only empirically observed.

### Amino acid/nucleotide metabolites

Branched chain amino acid metabolites and urea cycle metabolites were 2-to 2.9-fold higher in pasture-finished beef samples (Supplementary Table 1). This may be a further result of higher levels of physical activity in the pasture-finished animals, as findings are similar to the effects of exercise observed in other mammals, such as humans^34^. Carnitine and long-chain acyl carnitines, which are important for transport and metabolism of long-chain polyunsaturated fatty acids in the mitochondria^28^, as well as for maintaining diverse microbial populations in the gastrointestinal tract^35^, were 1.2- to 1.5-fold higher in pasture-finished beef. These findings further illustrate a more oxidative phenotype in pasture-finished cattle.

We also found various antioxidant and pro-oxidant amino acid metabolites to be differently altered because of finishing mode. In particular, we found that anserine—a derivative of carnosine with antioxidant capacity^36^—was 1.2-fold higher in grain-finished beef, while urate—another major intracellular antioxidant—was 2.6-fold higher in pasture-finished samples. 4-hydroxynonenal glutathione, an advanced lipoxidation end-product, was 2.6-fold higher in grain-finished samples. Advanced glycation end products (AGEs) and advanced lipoxidation end products (ALEs) are products of nonenzymatic reactions between reducing sugars and proteins, lipids, and/or nucleic acids. AGEs and ALEs as well as their precursors share common pro-inflammatory properties^37^, and higher dietary intakes have been implicated in a variety of metabolic diseases^38^. Other notable metabolites included homocysteine and *S*-adenosylhomocysteine, which were increased by 1.7- and 1.5-fold respectively in grain-finished beef. Elevated levels of these compounds are formed as products of impaired *S*-adenosyl methionine (SAM) cycle metabolism and Elevated levels of both metabolites are associated with vascular disease^39^ and considered markers of oxidative stress^40^. Taken together, these metabolites likely indicate increased antioxidant status and decreased oxidative stress levels in the pasture-finished cattle relative to grain-finished animals, which have higher levels of AGEs and ALEs. Whether consuming meat with different levels of AGEs and ALEs impacts metabolic disease risk of consumers is currently unclear but a potentially important area of future study.

While 4-hydroxynonenal glutathione, homocysteine, and *S*-adenosylhomocysteine were higher in grain-finished beef samples; potentially indicating increased oxidative stress in the feedlot animals, we observed 1.3-fold higher levels of oxidized glutathione and 0.6-fold lower levels of reduced glutathione in pasture-finished beef. Glutathione is a tripeptide that serves as an intracellular antioxidant vital to a wide variety of processes including hepatic detoxification and mitochondrial function^40^. The reason why glutathione was oxidized to a greater extend in the pasture-finished animals is not directly clear but is potentially explained by the greater transport time (~ 500-km transport for the pasture-finished animal as opposed to an on-site processing plant for the feedlot-finished animals), and resultant acute stress for the pasture-finished animals^41^.

### Vitamins and cofactors

**Fig. 6:**
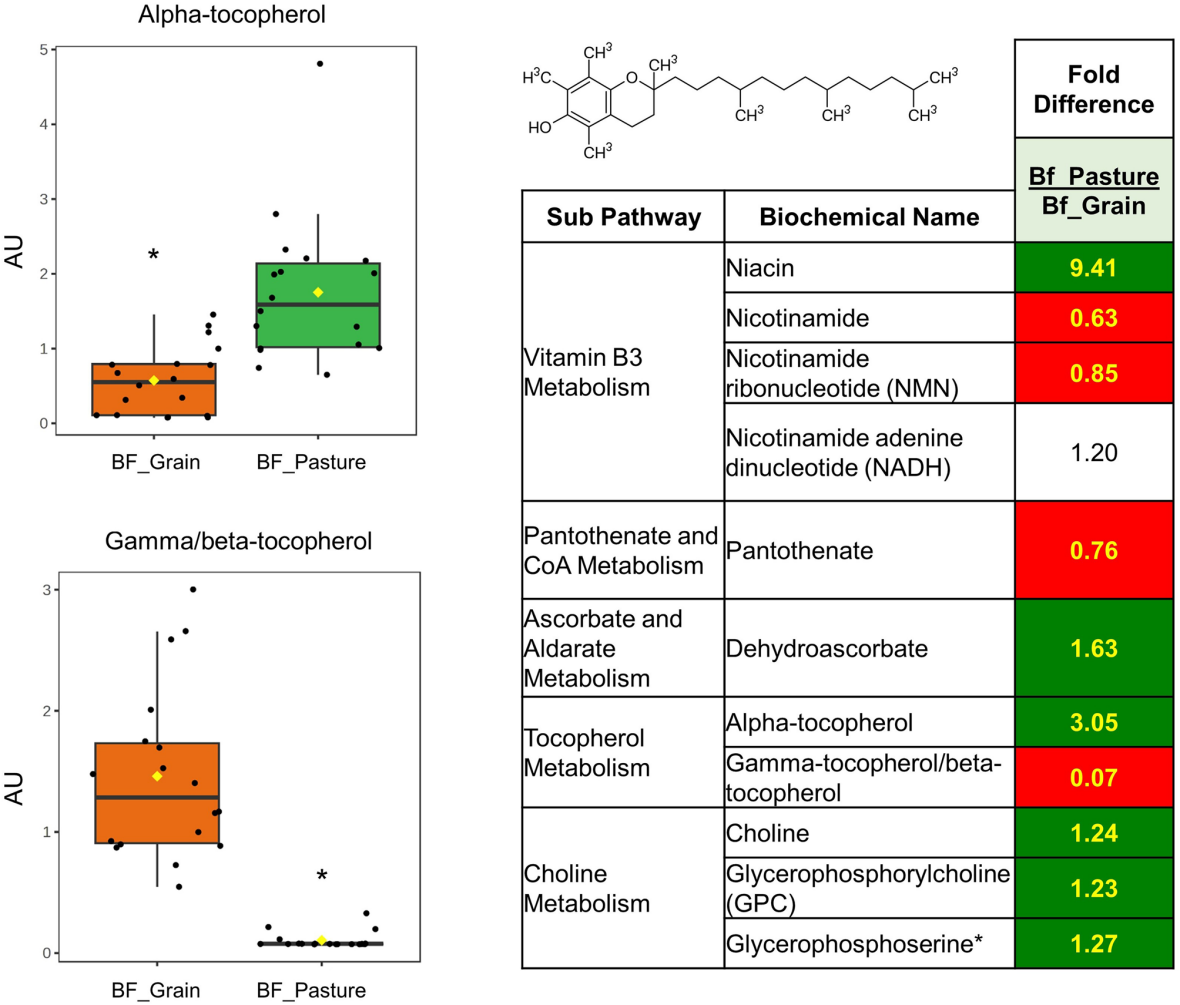
Alpha-tocopherol (a Vitamin E precursor), niacin (a form of Vitamin B_3_), choline, and dehydroascorbate (product of Vitamin C metabolism) were 3-, 9.4-, 1.2-, and 1.6-fold higher pasture-finished beef, respectively (all, *P* < 0.05). Alternately, pantothenate (Vitamin B5), gamma/beta tocopherol (a Vitamin E precursor), and nicotinamide (a form of Vitamin B_3_) were 1.3-, 14.6-, and 1.6-fold higher in grain-finished beef, respectively (all, *P* < 0.05). Values on figures are expressed as arbitrary units (AU).

Vitamins and vitamin precursors presented a more mixed picture across beef samples (Fig. 6). Consistent with previous literature on pasture-finished ruminants^8,42, 43^, α-tocopherol (a vitamin E precursor) was elevated threefold in pasture-finished beef, while γ/β tocopherol (also Vitamin E precursors) was elevated 14.6-fold in grain-finished beef. The observed differences likely reflect a greater content of α-tocopherol present in the diet of pasture-finished compared to grain-finished cattle. Fresh forages are rich in α-tocopherol, while grains typically contain higher amounts of γ-tocopherol^7^. Although γ-tocopherol is the most prevalent tocopherol in the US diet^38^ (γ-tocopherol is rich in plant seeds and their oils), α-tocopherol is the main tocopherol found in mammalian tissue and demonstrates the highest biological activity^44^. Nonetheless, γ-tocopherol has been characterized as a chemoprotective and pro-apoptotic compound, reducing reactive nitrogen species and reactive oxygen species in colon cancer models^45^.

Variation amongst B-vitamins was also observed. Niacin (Vitamin B_3_) was 9.4-fold higher in pasture-finished beef, while nicotinamide (the form of Vitamin B_3_ most used in supplements) was 1.6-fold higher in grain-finished beef. Pantothenate (Vitamin B5) was also elevated 1.3-fold in grain-finished beef, probably due to the relatively high amount of this vitamin in corn. Thus, feeding a limited amount of grain, or grain by-products (such as grazing machine-harvested crop fields that leaves some unharvested grain and plant residue or providing other edible industrial by-product residue) may have some benefit for the nutritional composition of meat. We also found dehydroascorbate (a Vitamin C metabolite) to be 1.6-fold higher in pasture-finished beef, likely reflecting its presence in fresh forages. Although ascorbate and dehydroascorbate were detected in ruminant muscle meat, meat is not considered a good source of Vitamin C.

Choline, an essential component of phosphatidylcholine that makes up 45–50% of all cellular membranes and the neurotransmitter acytlycholine^46^, was elevated 1.2-fold in pasture-finished beef. This nutrient is vital for the epigenetic regulation of gene expression and methylation reactions, most of which occur in the liver. While we found in higher concentrations in pasture-finished than grain-finished beef samples, ruminant meat is considered a rich source of choline in general regardless of finishing mode^1^.

### Phytochemicals/Xenobiotics

**Fig. 7:**
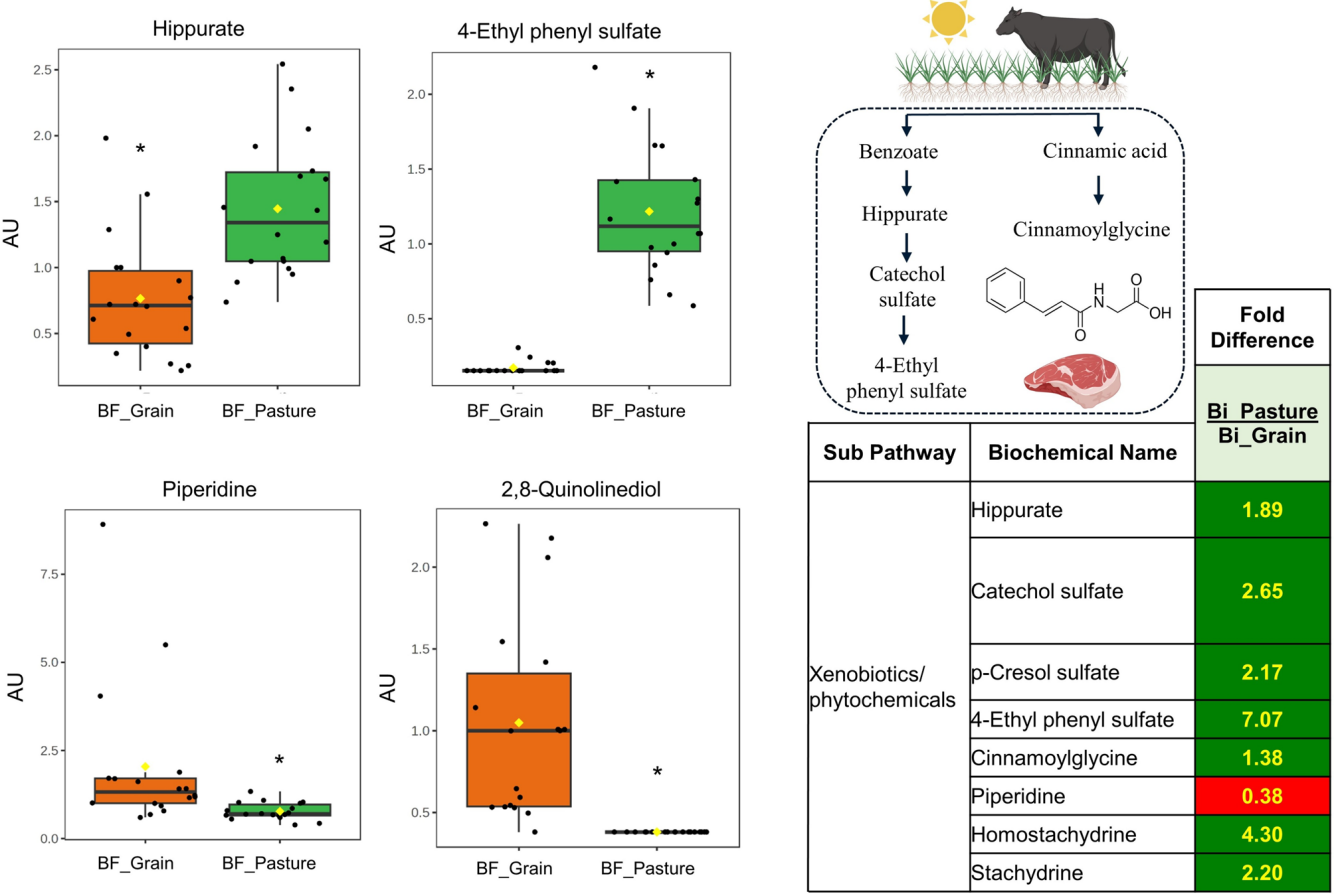
Hippurate and its downstream metabolites catechol sulfate and 4-ethylphenyl sulfate were 1.9- to 7.1-fold higher (all, *P* < 0.05). Cinnamoylglycine, a glycine conjugate of cinnamic acid, was 1.4-fold higher, while n-methylpipecolate, a downstream metabolic of the phenolic coumaric acid, was 4.9-fold higher (all, P < 0.05). Other phenolic metabolites elevated in pasture-finished beef included p-cresol sulfate, as well as stachydrine and homostachydrine (two major compounds derived from plants in the *Stachys* genus). Furthermore, 2,8-quinolinediol, a common downstream metabolite of the antibiotic fluoroquinolines, was only present in grain-finished beef samples (all, P < 0.05). Piperidine, and alkaloid commonly found in corn, was 2.6-fold higher in grain-finished beef samples (*P* < 0.05). Values on figures are expressed as arbitrary units (AU).

Fresh forages are typically rich in antioxidant compounds commonly referred to as phytochemicals. These plant-derived bioactive compounds include various phenolic metabolites, such as phenolic acids, flavonoids, tannins, and other plant-secondary metabolites studied for their wide-ranging roles in animal and human health^10^. We found a 2.6-fold greater total phytochemical content in pasture-finished beef, which is most readily explained by the cattle’s diet as the pasture-finished cattle in this work had access to over 500 grasses, forbs, and shrub species in Western U.S. rangelands from which they could choose their diet. Other factors (such as physical activity) can also impact the microbial populations in cattle rumen responsible for metabolizing plant phenols^47^, which further indicates that both the higher phytochemical diversity and physical activity in pasture-finished animals are likely responsible for many of the findings made in this work (Fig. 7).

Elevated phenolic metabolites included hippurate, which was 1.9-fold higher in pasture-finished beef, and its downstream metabolites catechol sulfate and 4-ethylphenyl sulfate, which were 2.7- and 7-fold higher, respectively. Hippurate is considered a biomarker of dietary phenolic intake in mammals^48,49^, and higher circulating levels of hippurate in humans without kidney disease are associated with improved metabolic health and diversity of the gut microbiome^50–52^. Cinnamoylglycine, a glycine conjugate of cinnamic acid, was elevated 1.4-fold in the pasture-finished beef. Cinnamic acid and its metabolites, which are dominant phenols found in plants, have potential anti-inflammatory effects^53^ and are linked to protective benefits related to various cancers^54^ and cognitive decline^55^. Stachydrine and homostachrydine, two major antioxidant compounds derived from plants in the *Stachys genus* (one of the largest in the mint family of Lamiaceae) were elevated by 2.2-fold and 4.3-fold respectively. This may be explained by the common occurrence of mint plants in North American rangelands^56^. Additionally, *N*-methylpipecolate, a downstream metabolite of coumaric acid, which reduces oxidative stress and tumor activity in colorectal cancer models^57^, was increased by 4.9-fold in pasture-finished beef. Hercynine (histidine betaine), a downstream metabolite of ergothioneine with potential antioxidant activities^58^, was 2.0-fold higher in pasture-finished beef. Ergothioneine and hercynine are produced by fungi and mycobacteria in soils, thus providing a potential connection with soil health^59^. Piperidine, an alkaloid with potential anti-oxidant and anti-cancer effects in vitro^60,61^, was 2.6-fold higher in grain-finished beef samples. While this compound is found to have antioxidant properties in human cell lines, some caution is warranted for livestock. Alkaloids such as piperidine have been implicated in alkaloid poisoning of cattle and horses were fed conserved forages^62^. As plants containing alkaloids in substantial amounts are generally avoided by cattle and horses, health issues to livestock usually occur when the plants contaminate conserved forages or when forage diversity is severely limited^62^. We do not know the source of the piperidine or whether the levels we observed are of any concern as our metabolomics analysis was not quantitative, but we consider the observation noteworthy.

While pasture-finished beef samples concentrated additional phytochemicals with ascribed health-promoting qualities, it is currently not known whether eating beef enriched in these phytochemicals can have an appreciable effect on human health, as few studies have been performed to determine potential anti-inflammatory and antioxidant effects of pasture-finished meat (and milk) consumption in humans^63–65^, and none have made a direct connection with the phytochemical richness of the ruminant’s diet. Given that our work focused on pasture-finished beef from cattle grazing in mountain rangelands in the Western U.S., our findings are not broadly generalizable to pasture-finished meat from cattle grazed in other areas of North America, as regional plant diversity is likely to impact the phenolic composition of meat, which is similar to findings made in dairy cows grazing mountain or lowland pastures in European dairy systems^66^. Nonetheless, several metabolites such as hippuric acid, p-cresol sulfate, and catechol sulfate are compounds that can be produced in mammalian metabolism from a wide range of individual phenolics and are likely to be somewhat independent of specific plant species^67^; however, for these to be produced endogenously, the plants consumed by animals will have to be rich in phenolic compounds as they serve as precursors. While the ration fed to the grain-finished livestock in this work used common feed ingredients such as corn, distiller’s grain, and grass-hay, the metabolome of grain-finished beef in this work is also not broadly generalizable to all grain-finished beef either as feed ingredients in total mixed rations are subject to change based on cost and availability^68^. Finally, 2,8-quinolinediol and 2,8- quinolinediol sulfate, two metabolites potentially derived from the frequently used antibiotic class known as fluoroquinolones^69^, were found in 94 and 100% of grain-finished beef samples, respectively. These compounds were not detected in the pasture-finished beef samples. Since the grain-finished meat came from animals not treated with antibiotics, as per personal communications with rancher supplying the feedlot-finished beef, we are unsure about their origin^70^. As a result, multi-residue veterinary drugs are often detected in distiller’s grain, and antibiotic residues present in distiller’s grain can be transferred to animal tissue upon ingestion^71^, which is likely what we observed in our work.

## Conclusion

We examined nutritional differences between pasture-finished and grain-finished beef from two commercial US-based farms. By profiling over 1500 compounds across multiple metabolic classes, our work serves as one of the most in-depth comparisons of beef from two US finishing systems (pasture-finished on Western rangelands and grain-finished in a feedlot) to date. Overall, our findings indicate that pasture-finishing of cattle (fed diverse species of grasses, forbs, and shrubs) concentrates additional compounds with ascribed health benefits and appears to improve metabolic health pathways compared to feedlot-finishing (fed total mixed rations rich in grains).

Our findings echo previous metabolomics findings in bison^33^ and beef^14^, and are in line with broader literature that pasture-finished beef contains a more favorable composition of very-long chain poly-unsaturated fatty acids (more omega-3 fatty acids) than grain-finished beef^5,7, 8^. We also observed enrichments in long-chain saturated fatty acids and long-chain acyl carnitines, which are associated with improvements in cardiovascular health. We also observed substantial differences in phytochemicals (plant-derived phenolics) as well as Vitamins B and E in the meat of pasture-finished and grain-finished cattle, which is likely reflective of their contents in the forage and feed, respectively. Specifically, alpha-tocopherol (Vitamin E precursor), Vitamin C, choline, and niacin (Vitamin B3) were increased in pasture-finished beef, while grain-finished beef contained higher amounts of gamma-tocopherol (Vitamin E precursor commonly found grains), nicotinamide (the form of Vitamin B3 most used in supplements), and pantothenate (Vitamin B5, which is also rich in grains). In terms of phytochemical antioxidants, we found that most of the measured compounds were increased in pasture-finished beef. While these compounds have antioxidant effects and benefit metabolic health both in vivo in in vitro, it is currently unknown whether their accumulation in meat has an appreciable effect on consumer health. Future randomized controlled trials in humans should be conducted to address this gap. Nonetheless their presence appears to have benefitted the animal’s metabolic health.

Pasture-finished beef samples indicated less oxidative stress and improved antioxidant capacity compared to grain-finished beef samples. We also observed a shift in cellular metabolism amongst pasture-finished beef samples with an increased capacity for oxidative metabolism and improved mitochondrial function, both of which could be considered indicative of improved cardiovascular health. Advanced glycation end products (AGEs) and advanced lipoxidation end products (ALEs) were elevated in grain-finished cattle and this finding may warrant future investigation to better understand their potential implications for consumers, as dietary intakes of ALEs and AGEs have been associated with increased risk of metabolic disease^38^.

It is important to note that the data does not necessarily imply that grain-finished beef is unhealthy to eat, as beef is a source of many vital nutrients in the US diet regardless of finishing mode^1,70^. Several randomized controlled trials with grain-finished beef, consumed as part of “healthy” diets (such as the Mediterranean, DASH, and BOLD Diet) have shown favorable effects on cardiometabolic health compared to participant’s habitual and/or control diets^71,72^. Nevertheless, understanding the potential nutritional differences in meat produced by different finishing modes is particularly important given the growing interest in agroecological grazing practices and considerations among consumers, both for environmental impacts and animal-welfare. The Intergovernmental Panel on Climate Change considers agroecological approaches in animal food production systems (such rotational grazing on biodiverse landscapes, as employed in the pasture-finished system in this work) as promising solutions to reduce environmental pressures, in addition to shifting towards more minimally-processed plant foods in the human diet^73^.

Although the implications of our work are limited by the small number of farms, herd size, cut of meat, cooking method (oven roasted), single breed of cattle, and non-quantitative (untargeted) metabolomics assessment, our findings encourage further investigation, especially given the depth of profiling and previously unrecognized differences between pasture-finished and grain-finished beef. This work can serve as a foundation for future studies, including a larger number of farms in diverse geographic regions and with a variety of cattle breeds and finishing diets, to assess the generalizability of our interpretations. Such studies should be performed in conjunction with controlled human feeding trials to make more direct connections between various production systems and their impacts on animal and human health.

## Methods

### Sample processing

This study evaluated beef samples from two commercial operations in the US that represent common finishing systems (rangeland-finished on grasses, forbs, and shrubs [pasture-finished] vs. feedlot-finished on a corn-based total mixed ration [grain-finished]). Since this work evaluated cattle from commercial ranches, and did not involve intervention by the research team, Institutional Animal Care and Use Committee (IAUAC) approval was not necessary. All analyzed samples (pasture-finished and grain-finished) were from cattle harvested in September–October of 2020 and had a Black Angus genetic background. The pasture-finished animals were between 25 and 27 months of age, while the grain-finished animals ranged from 18 to 22 months of age at time of slaughter. All cattle were processed in USDA-inspected slaughter facilities and the researchers worked with the producers to collect meat samples (*Pectoralis profundus*) from 18 individual animals (*n* = 18) per group. The *Pectoralis profundus* was selected as the cut due to the ease of sourcing these from separate animals in the harvest facilities. The pasture-finished beef samples were sourced from Alderspring Ranch in May, Idaho, USA which employs adaptive grazing practices during rearing and finishing. During the spring/summer, the animals were rotationally grazed through 70 square miles of certified organic mountain rangeland in the Salmon Challis National Forest, ID where they had access to over 500 grasses, forbs, and shrub species from which to choose their diets. During the fall and winter, the cattle grazed organic home ranch pastures and/or ate certified organic hay at Alderspring Ranch, located at the Pahsimeroi Valley. The hay was harvested primarily from meadows at Alderspring Ranch and contained an estimated 20–40 different plant species, with dominant ones being dandelion grass (*Taraxacum officinale*), orchard grass (*Dactylis glomerata*), Kentucky bluegrass (*Poa pratensis*), sanfoin (*Onobrychis viciifolia*), alfalfa (*Medicago sativa*), and clover (*Trifolium*). The pasture-finished cattle were processed by Stillwater Packing Company, Columbus, MT, after being transported in a livestock semi-trailer for ~ 500 km (6½ hours) under temperature conditions of ~ 15–22 °C in the Fall of 2020.

The grain-finished beef samples were purchased from a distributor in Northern South Dakota, USA, who procure cattle from local feeder/finishing operations located within ~ 300 km from Aberdeen, South Dakota. During the cow-calf and stocker phase, the cattle grazed on native pastures owned or leased by the feeder/finishing operations in Northern South Dakota, USA. The pastures contained an estimated 20–30 plant species, with dominant ones being big or sand bluestem (*Andropogon* spp.), crested wheatgrass (*Agropyron cristatum*), annual brome (*Bromus *spp.), blue grama (*Bouteloua *sp.) and clover (*Trifolium *spp.). During the finishing phase, the grain-finished cattle were kept in a feedlot located at the same feeding/finishing operation for ~ 130 days. While the exact composition of the total mixed ration fed to grain-fed cattle varied slightly between ranchers, and cannot be pinpointed exactly to the grain-fed beef samples studied in this work, the cattle were generally finished on a total mixed ration consisting of ground corn (~ 55–60%), distiller’s grain (~ 10–15%), grass hay/corn silage (~ 30%), vitamin/minerals pre-mix (~ 2.6%), and rumensin (~ 0.4%). The hay was harvested from the same pastures owned by feeder/finishing operations in Northern South Dakota, USA and thus had a similar plant composition as the pastures mentioned above. Depending on the location of the feeder/finisher, the grain-finished animals were transported in a livestock semi-trailer for 50–300 km (1–4 h) under temperature conditions of ~ 18–20 °C in the Fall of 2020.

Upon arrival at the lab, all meat samples were stored in a − 40 °C freezer and processed for analysis within 3 weeks of arrival. The meat samples were ground individually in a commercial meat grinder, and patties (112 g) were cooked in a commercial oven (175 °C) until the internal temperature of the patties registered at 71 °C as determined by a meat thermometer. Thereafter ~ 2 g from the center of each patty was obtained (*n* = 18 pasture-finished beef; *n* = 18 for grain-finished beef), immediately frozen in liquid nitrogen, and stored at – 80 °C until further analysis. Samples were subsequently analyzed for untargeted metabolomic and complex lipid profiling through collaborations with Metabolon (Morrisville, NC) and analyzed for fatty acid content at the Department of Nutrition, Dietetics, and Food Sciences, Utah State University (Logan, UT).

For our power calculations on sample size, we assumed a *P*-value ≤ 0.05, a *Q*-value ≤ 0.3, and a standard deviation of 0.2 based on previous work using untargeted metabolomics profiling of beef samples^14^. Eighteen (*n* = 18) meat samples per group were expected to provide true discovery rates ranging from 91 to 96% assuming differences in 200–240 metabolites.

### Metabolomics profiling

**Fig. 8:**
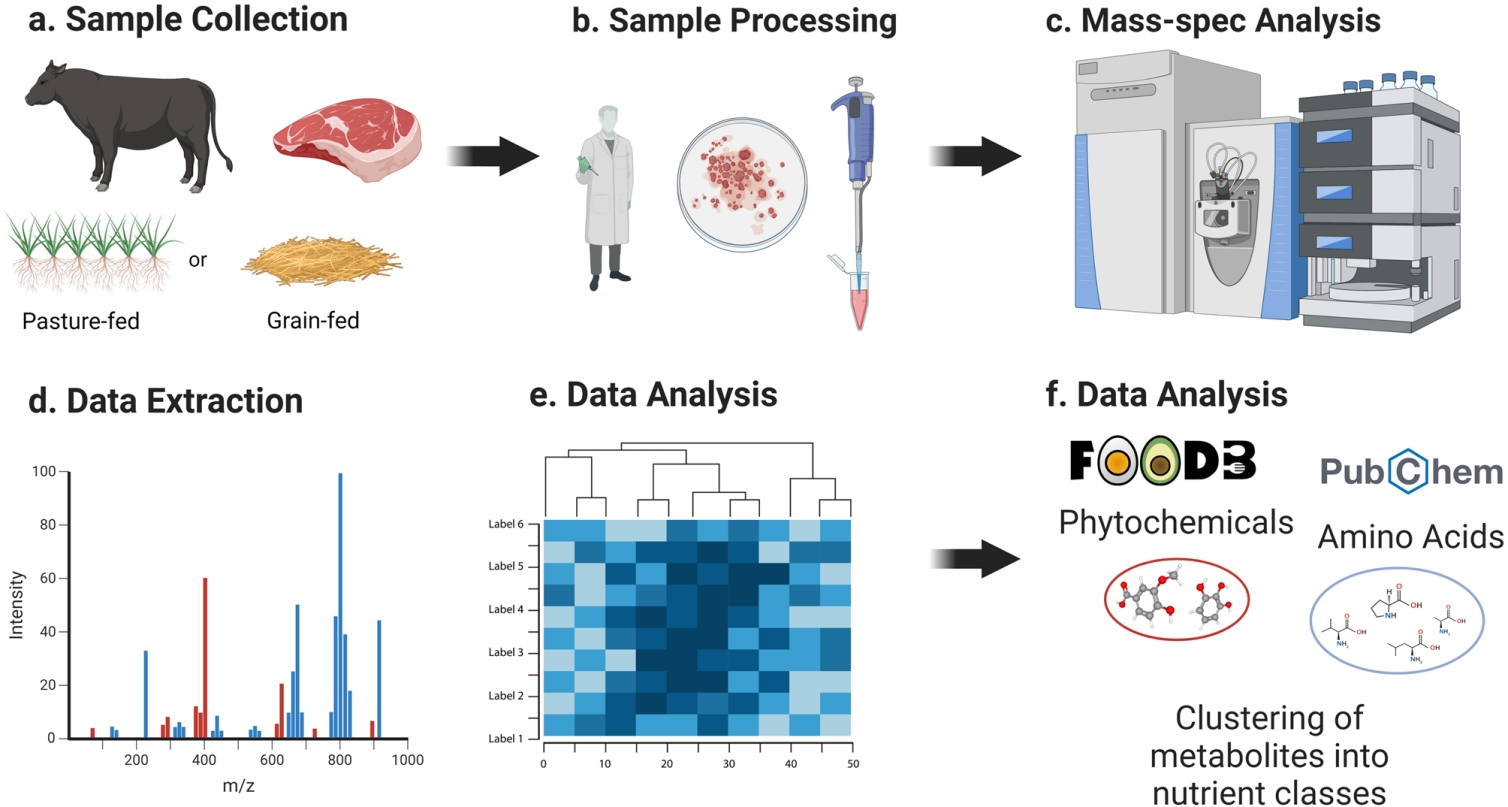
Schematic overview of sample collection and metabolomics analysis. (**a**) Cattle were finished on Western U.S. rangeland pasture (pasture-finished) or a U.S. Midwest commercial feedlot with total mixed ration consisting of corn, distillers’ grain, and hay (grain-finished). (**b**) Meat samples (*pectoralis profundus*) were cooked and ground, and extractions were performed using a MicroLab STAR system. (**c**) Subsequent metabolomic analysis was conducted via LC–MS/MS. (**d**) Metabolites were integrated using peak identification software, and (**e**) data was analyzed using Metabolon Pathway Explorer, ChemRICH, and Metaboanalyst. (**f**) Data interpretation, including potential bioactivities and health effects of identified metabolites, was aided by using FoodDB and PubChem.

A visual diagram of the study methodology is provided in Fig. 8. Sample preparation was carried out as described^33^. Briefly, 100 mg was weighed out for each sample and recovery standards were added for quality control purposes. Proteins were subsequently precipitated with methanol under vigorous shaking for 2 min (Glen Mills Geno Grinder 2000, Clifton, NJ, USA) followed by centrifugation (15,000×*g*). The resulting extract was divided into five fractions: two for analysis by separate reverse phase (RP)/UPLC-MS/MS methods with positive ion mode electrospray ionization (ESI), one for analysis by RP/UPLC-MS/MS with negative ion mode ESI, one for analysis by HILIC/UPLC-MS/MS with negative ion mode ESI, and one sample for backup. Sample extracts were placed briefly on a TurboVap (Zymark) to remove the organic solvent and reconstituted in mobile phases described below.

The UPLC-MS/MS platform utilized a Waters Acquity UPLC with Waters UPLC BEH C18-2.1 × 100 mm, 1.7 μm columns and a Thermo Scientific Q-Exactive high resolution/accurate mass spectrometer interfaced with a heated electrospray ionization (HESI-II) source and Orbitrap mass analyzer. One aliquot was analyzed using acidic positive ion conditions, which was chromatographically optimized for more hydrophilic compounds. The extract was gradient eluted from a C18 column (Waters UPLC BEH C18-2.1 × 100 mm, 1.7 µm) using water and methanol, containing 0.05% perfluoropentanoic acid (PFPA) and 0.1% formic acid (FA). The second aliquot was also analyzed using acidic positive ion conditions; however, it was chromatographically optimized for more hydrophobic compounds. The extract was gradient eluted from the same C18 column using methanol, acetonitrile, water, 0.05% PFPA and 0.01% FA.

The third aliquot was analyzed using basic negative ESI-optimized conditions using a separate dedicated C18 column. The basic extracts were gradient eluted from the column using methanol and water with 6.5 mmol/L Ammonium Bicarbonate at pH 8. The fourth aliquot was analyzed via negative ESI following elution from a HILIC column (Waters UPLC BEH Amide 2.1 × 150 mm, 1.7 µm) using a gradient consisting of water and acetonitrile with 10 mmol/L Ammonium Formate, pH 10.8. The MS analysis alternated between MS and data-dependent MS^n^ scans using dynamic exclusion, while the scan range covered *m/z* 70–1000 at a resolving power of R = 35,000 optimized at fifty percent of the maximum peak height (FWHM).

Metabolites were identified by automated comparison of the ion features in the samples to a reference library of chemical standard entries that considered the retention time, molecular weight (*m/z*), preferred adducts, in-source fragments, and associated MS spectra^74^. The data were curated by visual inspection for quality control using Metabolon’s proprietary software. Library matches for each compound were checked for each sample and corrected if necessary. Peaks were quantified using area-under-the-curve. A data normalization step was performed to correct for variation resulting from instrument inter-day tuning differences by setting the medians to equal one (1.00) and normalizing each data point proportionately (termed “block correction”). This preserved variation between samples while allowing metabolites of different raw peak areas to be compared on a similar graphical scale.

### Lipidomics profiling

One hundred (100 mg) was weighed out for each sample, which were soaked overnight in 1:1 dichloromethane:methanol at 4 °C. The supernatants were subjected to a Bligh–Dyer extraction^75^, using methanol/water/dichloromethane in the presence of deuterated internal standards. The extracts were concentrated under nitrogen and reconstituted in 0.25 mL of 10 mmol/L ammonium acetate dichloromethane:methanol (50:50). The extracts were transferred to inserts and placed in vials for infusion-MS analysis, performed on a Shimadzu LC (Canby, OR, USA) and a Sciex SelexIon-5500 QTRAP (Framingham, Massachusetts, USA). The samples were analyzed via both positive and negative ESI. The 5500 QTRAP scan was performed in multiple reaction monitoring (MRM) mode. Individual lipid species were quantified by taking the peak area ratios of target compounds and their assigned internal standards, then multiplying by the concentration of internal standard added to the sample. Lipid species concentrations were background-subtracted using the concentrations detected in process blanks (water extracts) and run-day normalized (when applicable). The resulting background-subtracted, run-day normalized lipid species concentrations were then used to calculate the lipid class concentrations.

### Fatty acid profiling

Fatty acid methyl ester (FAME) analysis was carried out as described previously with slight modifications^76^. One hundred mg of wet-weight sample was weighed into a screw-cap glass vial along with a 100 µL internal standard solution of tridecanoic acid (0.5 mg/mL in methanol; T-135; Nu-Chek Prep, Inc., Elysian, MN), 70 µL of 10 N KOH in water and 530 µL of methanol. The vial was subsequently sealed with a polypropylene-lined cap (ThermoFisher Scientific, Waltham, MA). Vials were placed in a shaking water bath (catalog number 67120; Precision Scientific, Chicago, IL) for incubation at 55 °C for 1.5 h. Samples were subsequently cooled on ice and 580 µL of 24 *N* H_2_SO_4_ was added. Samples were then vortexed at high speed for 60 s and then incubated with shaking at 55 °C for 1.5 h. One ml hexane was used to extract FAME before analysis by gas chromatography (GC). Separation of FAME was performed by a Shimadzu GC-2010 equipped with a HP-88 capillary column (100 m by 0.25 mm by 0.20 μm; Agilent Technologies, Palo Alto, CA) and a flame ionization detector (FID). The gas chromatograph was operated based on the conditions described previously^77^. The column head pressure was 195.6 kPa and the total flow rate was 129.1 mL/min (column flow: 2.47 mL/min; purge flow: 3.0 mL/min). One microliter of sample was injected with a split ratio of 50:1. The oven method was as follows: 35 °C held for 2 min, then increased to a temperature of 170 °C at a rate of 4 °C/min, then held for 4 min, then increased to a temperature of 240 °C at a rate of 3.5 °C/min, and then held for 7 min. Hydrogen was used as the carrier gas. The injector and FID were operated at 250 °C. Fatty acids were identified based on the similarity of retention-fold with GC reference standards (Nu-Chek Prep, Inc.). Fatty acid concentrations were calculated as a % relative to total fatty acids.

### Data analysis

Prior to statistical analysis, samples were normalized to mass, log transformed, and missing values were imputed with the minimum observed value for each compound. To test differences in individual metabolites between groups, non-parametric Wilcoxon rank sum *t*-test was performed using 5% as the cut-off for statistical significance (*P* < 0.05), while false-discovery rate statistics (q-values) were performed on the omics-data to account for multiple comparisons^78^. A Q-value (*Q* < 0.10) was considered to indicate high confidence in the *t*-test result for the given metabolite. Next, sparse PLS-DA (sPLS-DA) was performed to visualize data sets and identify the top 20 metabolites that discriminated between groups using Mean Decrease Accuracy (MDA) as the metric for order of importance. Subsequently, spearman rank correlations between log transformed data of the variables with PCA loadings > 0.60 and < − 0.60 (first principal component) were calculated for the pasture-finished and grain-finished beef samples separately. Correlations with values > 0.70 and < − 0.70 were visualized using Cytoscape3.9.1^79^. Correlations of the grain-finished beef samples were subtracted from the correlations of the pasture-finished beef samples, and values > 0.50 and < − 0.50 were visualized as a network as well. The perfuse force directed layout algorithm was used to generate the network topology^80^. Statistical analyses were performed in ArrayStudio/Jupyter Notebook, MetaboAnalyst (https://www.metaboanalyst.ca/) and R (http://cran.r-project.org/). Further cluster analysis was conducted using ChemRICH (https://chemrich.idsl.me/) software by ontology mapping and structural similarity using InChiKeys and SMILES as described^81^, and clusters with > 3 compounds were retained for analysis and reported. Annotated metabolites were investigated for potential health effects and bioactivities by entering the Chemical Abstracts Service (CAS) # of individual metabolites in PubChem (https://pubchem.ncbi.nlm.nih.gov/) and/or FooDB (https://foodb.ca/) databases, in addition to performing Google Scholar and PubMed searches by individual compound name to capture broader literatures. KEGG IDs^82^ were provided by Metabolon for presentation purposes in Supplementary Table 3. Various aspects of figures were created using Biorender (https://biorender.com/).

### Supplementary Information

Supplementary Table 1.

Supplementary Table 2.

Supplementary Table 3.

Supplementary Table 4.

## Data Availability

The datasets supporting the conclusions of this article are included within the article. In particular, the metabolomics and lipidomics data used for statistical analysis and interpretation to be found in Supplementary Tables 3 and 4, respectively. Chromatographic data files can be provided by the corresponding author upon reasonable request. The metabolomics data of this study has been deposited on Metabolomics Workbench: 10.21228/M8HC1Q. Metabolomics Workbench is supported by NIH grant U2C-DK119886.

## Abbreviations

AGE: Advanced glycation end product
ALE: Advanced lipoxidation end product
ALA: Alpha linolenic acid
DHA: Docosahexaenoic acid
DPA: Docosapentaenoic acid
EPA: Eicosapentaenoic acid
ESI: Electrospray ionization
FAME: Fatty acid methyl ester
FID: Flame ionization detector
GC: Gas chromatography
MRM: Multiple reaction monitoring
PCA: Principal component analysis
SAM: *S*-Adenosyl methionine
SFA: Saturated fatty acid
TAG: Triaglycerol
TCA: Tricarboxylic acid
RF: Random forest
RP: Reverse phase
UPLC-MS/MS: Ultra-high performance liquid chromatography tandem mass-spectrometry
US: United States
USDA: United States Department of Agriculture
